# Modulation of Radiation-Induced Genetic Damage by HCMV in Peripheral Blood Lymphocytes from a Brain Tumor Case-Control Study

**DOI:** 10.3390/cancers2020420

**Published:** 2010-04-12

**Authors:** Elizabeth A. Rourke, Mirtha S. Lopez, Claudia M. Monroy, Michael E. Scheurer, Carol J. Etzel, Thomas Albrecht, Melissa L. Bondy, Randa A. El-Zein

**Affiliations:** 1 Department of Epidemiology, The University of Texas M.D. Anderson Cancer Center, Houston, TX 77030, USA; E-Mails: erourke@mdanderson.org (E.A.R.); mirlopez@mdanderson.org (M.S.L.); cmonroy@mdanderson.org (C.M.M.); cetzel@mdanderson.org (C.J.E.); 2 Department of Pediatrics and Dan L. Duncan Cancer Center, The Baylor College of Medicine, Houston, TX 77030, USA; E-Mail: scheurer@bcm.edu (M.E.S.); 3 Department of Microbiology and Immunology, The University of Texas Medical Branch, Galveston, TX 77555, USA; E-Mail: talbrech@utmb.edu (T.A.)

**Keywords:** HCMV, brain tumors, CBMN-CYT assay, chromosome aberrations

## Abstract

Human cytomegalovirus (HCMV) infection occurs early in life and viral persistence remains through life. An association between HCMV infection and malignant gliomas has been reported, suggesting that HCMV may play a role in glioma pathogenesis and could facilitate an accrual of genotoxic damage in the presence of γ-radiation; an established risk factor for gliomas. We tested the hypothesis that HCMV infection modifies the sensitivity of cells to γ-radiation-induced genetic damage. We used peripheral blood lymphocytes (PBLs) from 110 glioma patients and 100 controls to measure the level of chromosome damage and cell death. We evaluated baseline, HCMV-, γ-radiation and HCMV + γ-radiation induced genetic instability with the comprehensive Cytokinesis-Blocked Micronucleus Cytome (CBMN-CYT). HCMV, similar to radiation, induced a significant increase in aberration frequency among cases and controls. PBLs infected with HCMV prior to challenge with γ-radiation led to a significant increase in aberrations as compared to baseline, γ-radiation and HCMV alone. With regards to apoptosis, glioma cases showed a lower percentage of induction following *in vitro* exposure to γ-radiation and HCMV infection as compared to controls. This strongly suggests that, HCMV infection enhances the sensitivity of PBLs to γ-radiation-induced genetic damage possibly through an increase in chromosome damage and decrease in apoptosis.

## 1. Introduction

Malignant gliomas are the most common brain tumors in adults and are rapidly fatal [1,2]. The American Cancer Society estimated that 22,070 new cases of primary brain and central nervous system (CNS) tumors were diagnosed in 2009 and approximately 12,920 cases died from the disease [3]. Approximately 77% of CNS tumors are brain gliomas [4,5] while half of all gliomas are glioblastoma multiforme (GBM); the most fatal histological type. Currently, little is known about the pathogenesis of gliomas or the possible contributions of environmental factors to the oncogenic process. Several studies have reported the effects of dysregulated cellular pathways in GBM development; in particular, alterations in cell proliferation and cell-cycle control resulting in apoptosis seem to be important pathways [4,5].

Epidemiological studies have explored the association between genetic alterations induced by ionizing radiation and glioma development [6,7]. Ionizing radiation has been shown to cause chromosomal aberrations, mainly double strand breaks, which facilitate an ongoing cycle of DNA damage, dysregulation of cellular pathways and overall genomic instability [8,9,10]. The DNA alterations caused by radiation are thought to be the promoters of malignant glial cell transformation and development. Bondy *et al*. [11,12,13] and Lui *et al*. [14], reported that exposure to ionizing radiation was consistently observed as an independent risk factor for brain tumors with higher levels of chromosome damage, with an increased sensitivity to radiation in glioma patients compared to controls. Exposure to therapeutic high-dose radiation has also been shown to increase risk of gliomas [4,15]. Studies by Edick *et al*. [16] and Relling *et al*. [17], have concluded that exposure to therapeutic cranial radiation is a likely risk factor for the development of secondary brain tumors of glial origin in cancer survivors. 

Recently, human cytomegalovirus (HCMV) was reported to increase risk of a number of cancers including prostate and cervical carcinomas, Kaposi’s sarcoma and gliomas [1,18]. HCMV is a herpesvirus that affects up to 80% of adults (depending on the source population), however, most infections remain subclinical [19,20]. After infection, the virus may enter into a latency period that can persist for years and be reactivated in response to immunosuppression or genotoxic exposures, which allows the virus to actively initiate mutagenesis through the disruption of cellular pathways, such as apoptosis and cell proliferation [21,22,23]. HCMV has been reported to induce genetic alterations that can potentially enhance the genotoxicity of environmental factors (radiation, chemicals, *etc.*) [24]. The oncogenic properties may also stem from the ability of HCMV to transactivate proto-oncogenes, which results in mutations in these normal cells that can then lead to cancer development [22,25]. Furthermore, our research has detected HCMV DNA and antigens present in glioblastoma, anaplastic and low-grade glioma tissues [23].

When combined with DNA damaging agents, such as ionizing radiation, HCMV’s oncogenic potential is enhanced [22,25]. Ohagen *et al*. [26], demonstrated the ability for γ-radiation to induce latent HCMV activation and expression. Similar studies focusing on pancreatic cancer have also found a significant association with radiation and the activation of HCMV promoters and virus replication [27]. Activated HCMV can induce chromosomal damage with subsequent genetic instability that facilitates the development of cancer. The mechanism for virus replication has been difficult to ascertain [24] but new *in vitro* testing techniques have provided insight into modes of replication and inhibition of HCMV [28,29] and furthermore, it has been shown to replicate in the presence of radiation [30]. In addition, AbuBakar *et al*. [31], has provided evidence illustrating HCMV’s ability to induce chromosomal aberrations when activated in the body. These aberrations, when coupled with exposure to genotoxic agents such as ionizing radiation, are hypothesized to increase the risk of malignancies at the site of infection [8]. 

Cancer is the result of multiple genetic changes that can be mediated through gross chromosomal changes, which have the potential to be cytogenetically detectable [32]. In the current study, we evaluated the modulation of radiation-induced genomic damage by HCMV using the CBMN-Cyt assay. The CBMN-Cyt assay is a widely used cytogenetic method to measure DNA damage by (1) scoring chromosome fragments or whole chromosomes that form micronuclei (MN) as a result of not engaging with the mitotic spindle and (2) nucleoplasmic bridges (NPBs) which originate from dicentric chromosomes whose centromeres are pulled to opposite poles of the cell at anaphase[33,34]. This technique is a comprehensive means of scoring cellular events in the form of not only chromosome damage, but also cell death. Thus, the CBMN-Cyt provides a better understanding of the underlying mechanisms involved in the sensitivity of cells to genotoxic exposures. MN have been used as a biomarker of cancer risk in lung and breast cancers [35,36,37,38,39]. This assay is an ideal means for evaluating the effects of chromosome damage modulated by HCMV and radiation; since it can identify and score an optimal amount of damage resulting from different chromosomal changes (*i.e.*, MN, NPBs and apoptosis). 

In the present study, we determined the effect of HCMV infection on modulating the level of radiation-induced chromosomal instabilities in PBLs of glioma patients and controls. 

## 2. Materials and Methods

### 2.1. Study Subjects

Cases (n = 110) were newly diagnosed subjects with histopathologically confirmed malignant gliomas, who were previously untreated and registered at the M.D. Anderson Cancer Center (MDACC). The cases were histologically classified as glioblastoma (n = 57) and other glioma (n = 53). Healthy controls (n = 100) were accrued through random-digit dialing and were frequency matched to cases on age (±5 years), gender and race/ethnicity. Initially, 110 controls were selected, but for matching purposes 10 were eliminated on the basis of ethnicity. The 110 cases were all maintained due to the rarity of gliomas. Detailed methods for recruitment of cases and controls are described by Liu *et al*. [14]. All cases and controls were residents of Harris County and willing to donate 30 mL of blood. Participants were asked to sign a consent form and provide information on medical and family history of cancer, occupational history, specific exposures, including smoking and alcohol use history. Information was also obtained from a review of the patient’s hospital records. Peripheral blood was obtained from all participants. This study followed a protocol approved by the Institutional Review Board of MDACC. 

### 2.2. Culture Preparation for Genomic Instability Measurements

Standard cytogenetic procedures were used to establish PBL cultures [40]. Aliquots of blood (0.5 mL each) were cultured in 4.5 mL RPMI-1640 medium (Gibco, Invitrogen, Carlsbad, CA, USA) supplemented with 10% fetal bovine serum (FBS) and 0.39 g/L glutamine. PBLs were stimulated to proliferate with the addition of phytohemagglutinin (PHA; 0.18 mg/ml; Remel, Lenexa, KS). For each individual, four cultures were prepared in duplicate for the CBMN-Cyt assay to determine the genomic instability level for the following conditions: baseline, radiation-induced, HCMV-induced, and both HCMV- and radiation-induced. Previously reported standardized procedures were used for HCMV treatment conditions and duration [8,9,24,40]. 

### 2.3. Cultures Infected with HCMV

HCMV, strain AD169, was selected for this study. Twenty-four hours after PHA stimulation, sedimentation was used to separate culture fluids and supernatant was removed and set aside for later use. In order to obtain a calculated multiplicity of infection (MOI) of approximately five plaque-forming units (PFU)/cell, PBLs were re-suspended in virus stock. The cells were incubated at 37 °C for 2 hours in order to sensitize the cells without overtaking cellular machinery or damaging cell viability. Once again, sedimentation was used to collect cells and the virus inoculum was removed and discarded. Next, the reserved supernatant was used to re-infect the cells at 37 °C. Cytochalasin B was added at 44 hours and cultures were harvested at 72 hours. In order to perform a mock-infection, cells were re-suspended in virus stock from which the virus had been removed and subsequently treated in the same manner as those infected with virus [20,41]. 

### 2.4. Cultures Treated with Radiation

The cells were radiated with γ-radiation from a 137 Cs source (Cesium Irradiator Mark 1, model 30; J.L. Shepherd and Associates, Glendale, CA, USA) 24 hours after PHA stimulation. Since the dose for a fractionated treatment in the clinic is generally 1.2–2 Gy/fraction, a dose of 1.5 Gy was used. Cells were then re-incubated at 37 °C. Cytochalasin B was added at 44 hours and cultures were harvested at 72 hours. 

### 2.5. Cultures Infected with HCMV and Treated with Radiation

Cells were infected with HCMV as described in Methods Section 2.3 Following this, the PBLs were washed twice with un-supplemented RPMI media, re-suspended in the reserved growth medium and then radiated with 1.5 Gy of γ-radiation (as described in Section 2.4). Cells were re-incubated at 37 °C. Cytochalasin B was added at 44 hours and cultures were harvested at 72 hours. 

### 2.6. CBMN-Cyt Assay Evaluation

Harvesting of cells, hypotonic treatment, fixation and slide preparation were all performed according to standard methods outlined by Fenech *et al*. [34]. Fixed cells were dropped onto clean microscopic slides, air-dried and stained with Giemsa. Slides were coded for blinding to the reader. For each sample, 1,000 binucleated cells were scored using a Nikon E-400 microscope following the criteria outlined by Fenech *et al*. [33]. The micronuclei (chromosomal breaks) and nucleoplasmic bridges (chromosomal rearrangements) frequencies in 1,000 binucleated cells were recorded. Nuclear bud frequencies in 1,000 binucleated cells were also scored; however, the frequencies were very low and therefore not maintained in the results. Slides obtained from different batches were all scored together. In addition, 500 binucleated cells were scored for frequency of apoptosis following the criteria outlined by Fenech *et al.* [33,34], in all experimental conditions: baseline, after treatment with radiation, HCMV and HCMV + radiation. This criteria is based on a morphological evaluation of apoptotic cells specifically those cells expressing a higher staining intensity of the cytoplasm, nuclear fragments and nucleus. Quality control measures were implemented by randomly selecting 20% of the slides to be blindly rescored. These values were compared with the original scores to verify consistency.

### 2.7. Statistical Analysis

Demographic characteristics were summarized and differences by case-control status were compared using Student’s t-test (if continuous) or the chi-squared test (if categorical). A one-way analysis of variance (ANOVA) was used to determine differences in mean values by case-control status and by treatment type. Bonferroni correction of p-values was used to avoid alpha problems with multiple post hoc tests. P-values were deemed significant at the <0.05 level. Mean levels for each treatment were adjusted for baseline measures using a univariate linear model. Paired t-tests, for more precise measures, were used to determine differences between treatment types for MN, NPBs and apoptosis among cases and controls (separately). All statistical analyses were performed with Intercooled Stata version 10.0 (Stata Corp., College Station, TX, USA) and SPSS, version 16.0 (SPSS Inc., Chicago, IL, USA).

## 3. Results and Discussion

### 3.1. Subject Characteristics

This study consisted of 210 participants (glioma cases = 110 and healthy controls = 100). The distribution of age, gender and race was not statistically significant between cases and controls. The mean age of the cases was 48.6 years and 50.5 years for controls (p = 0.216). Among the cases, 37% were male and 63% female and among the controls, 44% were male and 56% female (p = 0.321). Race was initially categorized as Caucasian, Hispanic and Asian however; the frequency of Hispanics and Asians was low and therefore, collapsed into one group classified as “others”. The case and control groups were 94% Caucasian and 6% other (p = 0.188). Histology was recorded for cases only and was comprised of 51% glioblastomas (GBMs) and 49% others (15% astrocytoma, 7% mixed glioma, 23% oligodendroglioma 3% ependymoma and 1% gangliocytoma). 

### 3.2. Baseline Chromosome Damage Frequencies

**Figure 1:**
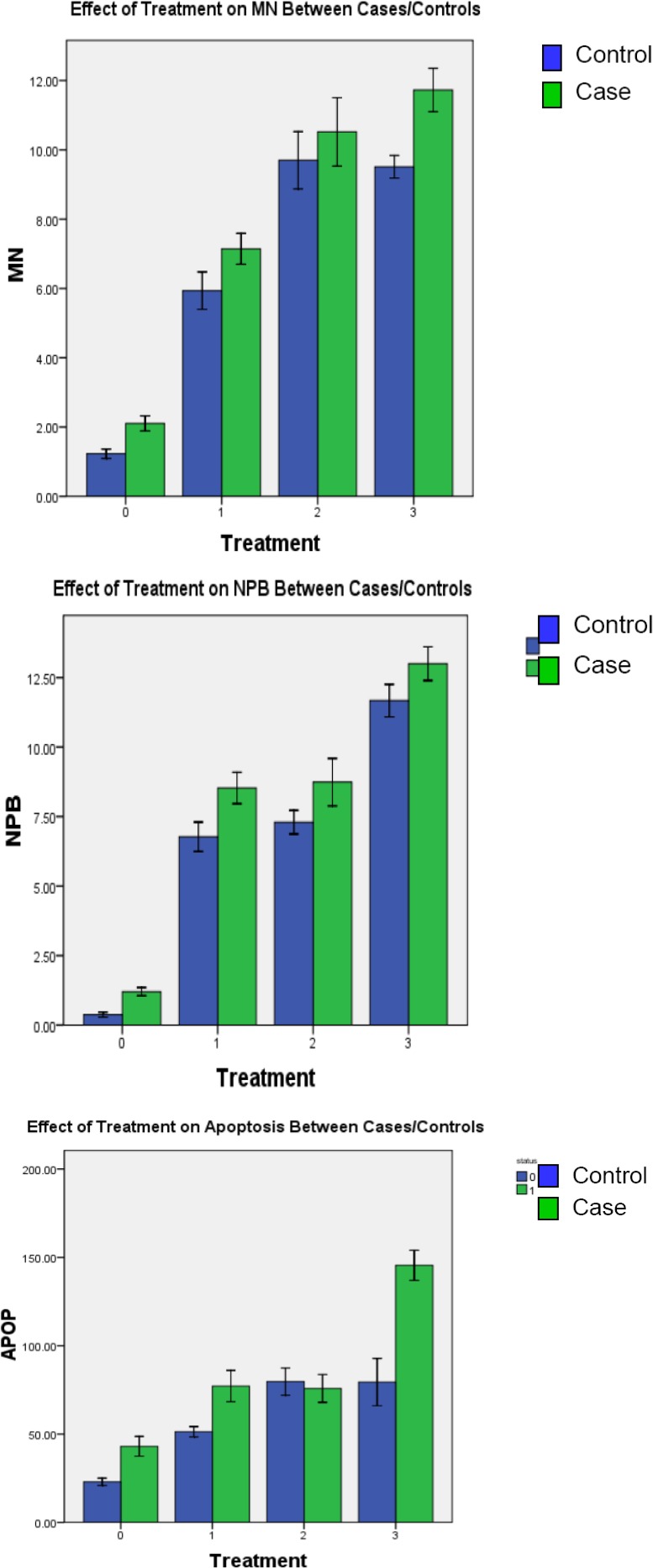
Relationship between induced chromosome damage (MN and NPBs) and apoptosis. The relationship between induced chromosome damage (A & B) and apoptosis (C) in cases and controls is measured and compared after each treatment of PBLs. Treatments: baseline = 0, radiation = 1, HCMV = 2 and HCMV + radiation = 3. These graphs depict the effects of treatments on genetic damage by presenting the difference between cases and controls, as well as within each group. Cases are identified by green bars and controls by blue bars. Almost consistently across the board, cases experience a greater frequency of overall induced genetic damage.

The baseline chromosome aberration frequency was significantly higher among cases when compared to controls for both MN and NPBs (p < 0.01). The mean ± S.E.M for MN in cases was 2.1 ± 0.11 and 1.2 ± 0.07 for controls, and 1.2 ± 0.07 and 0.37 ± 0.04 for NPBs of cases and controls, respectively (Figure 1). 

Analysis was conducted using SPSS 16.0. A one-way ANOVA was used to test for significance between cases and controls in each treatment group. P-values significant at the <0.05 level. Error bars represent the S.E.M. All of the groups were significant except for HCMV apoptosis (Figure 1, C Bar 2) and MN (Figure 1, A Bar 2).

### 3.3. Induced Chromosome Damage Frequencies

All experimental treatments were compared to baseline levels of MN and NPBs among cases and controls separately. All comparisons in cases and controls resulted in significant p-values at <0.01. Levels of induced damaged for radiation, HCMV and HCMV + radiation were adjusted for using baseline measures in a univariate analysis. Therefore, we can illustrate the true level of induced damage beyond initial baseline levels. It should be noted that the frequency of MN and NPBs for mainly cases at baseline and after radiation were on the lower end of the scale of what has been shown in other studies however; it is common for these frequencies to fluctuate based on laboratories and should not be discounted [10,36,38,42]. 

#### 3.3.1. Radiation-Induced Chromosome Damage

Radiation induced a significantly higher frequency of MN in cases (mean ± SEM = 7.1 ± 0.22) as compared to controls (5.9 ± 0.27; p < 0.01). Similarly, a significantly higher frequency of NPBs was observed in cases as well (8.5 ± 0.28) when compared to controls (6.8 ± 0.26; p < 0.01). Overall, cases experienced a higher induction of chromosomal damage following exposure to gamma radiation relative to baseline than the controls, which indicates that cases are more sensitive to genotoxic damage.

#### 3.3.2. HCMV-induced Chromosome Damage

PBL infection with HCMV induced a significantly higher frequency of MN in cases and controls than baseline measures (p < 0.01), however, there was no significant difference in the distribution of MN between cases and controls (mean ± SEM = 10.5 ± 0.49 and 9.7 ± 0.41, respectively). Significantly higher frequencies of NPBs were observed in cases as compared to controls when treated with HCMV (mean ± SEM = 8.7 ± 0.43 and 7.3 ± 0.21, respectively p = 0.004).

#### 3.3.3. HCMV + Radiation–induced Chromosome Damage

PBL infection with HCMV followed by γ-radiation induced a significantly higher frequency of MN in cases relative to controls (mean ± SEM = 11.7 ± 0.31 and 9.5 ± 0.16, respectively). Significantly higher frequencies of NPBs were also observed in cases as compared to controls (mean ± SEM = 13.0 ± 0.30 and 11.7 ± 0.29 respectively; p = 0.001).

Table 1 shows the analysis of repeated measures (paired t-tests) used to determine the difference between each treatment on MN and NPBs in cases and controls. Cases and controls both showed statistically significant differences (p-values < 0.01) in MN frequencies between radiation *vs*. HCMV and radiation *vs*. HCMV + radiation treatments. The results indicate that a higher frequency of chromosomal damage in terms of MN was seen in cells with HCMV and HCMV + radiation when compared to radiation alone. In addition, only the cases showed significant differences in MN when HCMV was compared to HCMV + radition (p < 0.01). This same comparison of HCMV *vs.* HCMV + radiation did not induce a significantly higher frequency of MN in controls. When NPB frequencies were compared in each treatment, the radiation *vs*. HCMV + radiation (p < 0.01) and HCMV *vs*. HCMV + radiation (p < 0.01) groups for both cases and controls, showed significantly higher levels of NPB induction. However, only controls showed a significantly higher frequency of NPBs in the radiation *vs*. HCMV treatment level (p = 0.036).

**Table 1:** Comparison between Treatments among Cases and Controls (Mean ± SEM).

	**Controls** Mean ± SEM	**Cases** Mean ± SEM
**Micronucleus **		
Baseline^b^	1.23 ± 0.07	2.10 ± 0.11
Radiation	5.94 ± 0.27	7.14 ± 0.22
Virus	9.70 ± 0.41	10.52 ± 0.49
Virus + Radiation	9.51 ± 0.16	11.73 ± 0.31
**Paired T-test**	**p-value^a^**	**p-value^a^**
Radiation *vs*. Virus	** <0.01**	** <0.01**
Radiation *vs*. Virus + Radiation	** <0.01**	** <0.01**
Virus *vs*. Virus + Radiation	0.631	** <0.01**
**Nucleoplasmic Bridges**		
Baseline^b^	0.37 ± 0.04	1.20 ± 0.07
Radiation	6.78 ± 0.26	8.53 ± 0.28
Virus	7.30 ± 0.21	8.74 ± 0.43
Virus + Radiation	11.67 ± 0.29	13.00 ± 0.30
**Paired T-test**	**p-value^a^**	**p-value^a^**
Radiation *vs*. Virus	**0.036**	0.647
Radiation *vs*. Virus + Radiation	** <0.01**	** <0.01**
Virus *vs*. Virus + Radiation	** <0.01**	** <0.01**
**Apoptosis**		
Baseline^b^	23.00 ± 1.03	43.04 ± 2.80
Radiation	51.32 ± 1.47	77.17 ± 4.42
Virus	79.71 ± 3.84	75.83 ± 3.92
Virus + Radiation	89.02 ± 5.86	143.95 ± 3.46
**Paired T-test**	**p-value^a^**	**p-value^a^**
Radiation *vs*. Virus	** <0.01**	0.736
Radiation *vs*. Virus + Radiation	** <0.01**	** <0.01**
Virus *vs*. Virus + Radiation	**0.012**	** <0.01**

*^a^* P-values significant at the <0.05 level. ^b^ All comparisons of baseline MN, NPBs and Apoptosis to Radiation, HCMV and HCMV + Radiation for cases and controls resulted in significant p-values at *<***0.01**.

### 3.4. Baseline and Induced Cell Death

Case and control frequencies of apoptosis were compared at baseline, after radiation induction, HCMV infection and HCMV + radiation induction. The frequency of apoptosis in cases was significantly higher at baseline, after radiation and HCMV + radiation than in controls. However, HCMV induced a higher frequency of apoptosis in controls as compared to cases. 

Table 1 also presents the results of paired t-tests utilized to determine the effects of each treatment on cell death among cases and controls. In cases and controls, apoptotic frequencies were significantly different in radiation *vs*. HCMV + radiation (cases: 77.17 ± 4.42 *vs.* 143.95 ± 3.46 p < 0.01 controls: 51.32 ± 1.47 *vs*. 89.02 ± 5.86 p < 0.01) and in HCMV *vs*. HCMV + radiation treatments (cases: 75.83 ± 3.92 *vs*. 143.95 ± 3.46 p < 0.01; controls: 79.71 ± 3.84 *vs*. 89.02 ± 5.86 p = 0.012). When radiation-induced apoptosis was compared to HCMV-induced apoptosis, only control frequencies were significantly different (51.32 ± 1.47 *vs.* 79.71 ± 3.84 p < 0.01). There was no significant difference in the frequency of apoptosis in samples treated with radiation alone or virus alone among cases. Furthermore, cases experienced nearly a 2-fold increase (75.8 ± 3.92 to 144.0 ± 3.46, p < 0.01) in apoptotic cells when HCMV alone was compared to radiated HCMV-infected cells, whereas controls experienced a much slighter (albeit significant) increase (79.7 ± 3.84 to 89.0 ± 5.86, p = 0.012).

### 3.5. Discussion

In the current study, we used the comprehensive CBMN-Cyt assay to evaluate the effect of HCMV infection on modulating the sensitivity of cells in response to exposure to radiation. This assay is slowly replacing conventional cytogenetics since it has the ability to restrict measurement to once-divided binucleated cells. More specifically, the CBMN-Cyt eliminates confounding effects of variability in kinetic cell division by allowing only those cells that have undergone nuclear division to be scored [42]. MN frequency, as established by the Human Micronucleus Project (HUMN), has been shown to be prospectively associated with an increased cancer risk and therefore can be used as a pathological biomarker for cancer. [10,33]. At baseline, cases displayed a significantly higher level of spontaneous genetic damage (MN and NPBs) when compared to controls. This increased level of chromosomal damage was also observed at radiation MN/NPB and HCMV NPB levels. Notably, our results showed a significant increase in MN and NPBs between baseline and radiation in both cases and controls; thus, substantiating our previous findings [12], using the metaphase-based mutagen sensitivity assay. Radiation induced genomic instabilities are also generated by dysregulation of the cell cycle resulting in apoptosis and cell proliferation [9,43]. Our findings are consistent with the notion of differential sensitivity to genotoxic agents in cancers, where the number of apoptotic cells present at baseline and after radiation were significantly higher in cases than in controls. Although intriguing, this observation could potentially by explained by the increased oxidative burden in the cancer cases. Oxidative stress-generated reactive oxygen species (ROS) are known to induce cellular senescence and apoptosis [44,45]. Even though the cases had a higher frequency of apoptotic cells than the controls, the induction of apoptosis was lower in cases than in controls, indicating the possibility of defective apoptotic machinery in the cases, thus allowing for the survival of damaged cells (Table 1).

Another notable finding is that for both cases and controls, the number of apoptotic cells was significantly increased following radiation (in reference to baseline measures). This validates previous studies that have indicated the capabilities of ionizing radiation to disrupt the cell cycle and ultimately induce apoptosis [46,47,48].To our knowledge, this is the first study to show that the HCMV-induced genetic damage is as high as that caused by radiation. It has been proposed that viral interactions may also play a role in arrested or impaired DNA repair capacity and subsequent tumorigenesis [22,49]. HCMV facilitates the accumulation of DNA damage in infected cells, when coupled with genotoxic insult, and has been shown to amplify chromosomal damage and overall genomic instability [8,41,50]. We therefore examined the modulating effect of HCMV infection on genomic instabilities in PBLs treated *in vitro* with γ-radiation. Our results showed a statistically significant difference between MN, NPBs and apoptosis at baseline and after HCMV infection followed by radiation treatment of PBLs for both cases and controls. When cases and controls were compared at baseline and after HCMV + radiation treatment, there were significantly higher numbers of MN, NPBs and apoptosis in cases. Several relationships of notable importance could be elucidated from these results. First, the effect of HCMV alone led to the induction of chromosome breaks (MN) to an extent similar to that induced by radiation, a known risk factor for brain tumors. Second, the frequency of MN increases significantly when PBLs were HCMV-infected prior to γ-radiated as compared to virus- or radiation-treatment separately, and the effects observed were higher in the cases than controls. This effect, in cases only, was less than additive but still a significant increase in HCMV + radiation induced MN as compared to radiation (7.14 *vs.* 11.73) and virus (10.52 *vs.* 11.73) alone. There are a number of plausible explanations to such an observation: first, HCMV infection may cause the cell to be more sensitive to damaging effects of genotoxic agents; and second, the genetic damage observed is a result of accumulation of HCMV-induced damage plus that induced by the genotoxic exposure. While chromosome breaks (in the form of MN) tend to be more significantly modulated by HCMV and radiation in cases, chromosome rearrangements (NPBs) seem to be less affected by disease status and are similar in cases and control groups. NPBs are significantly increased in cases and controls when HCMV and radiation are coupled, but not to the extent of an additive effect; rather, the observed increase is predominantly due to the effect of radiation. 

HCMV encodes gene products that can result in cell proliferation, dysregulation and alteration of apoptosis [24,41,51]. The current study sought to evaluate the modulating effect of HCMV, radiation and both treatments on apoptosis in PBLs. Interestingly, despite a higher level of apoptosis at baseline in the cases, the induction of apoptosis occurred to a lower extent following radiation (1.8- *versus* 2.2-fold), HCMV infection (1.8- *versus* 3.5-fold) and HCMV infection + radiation (3.3- *versus* 3.9-fold) in cases compared to controls. In addition, our results also suggest that further challenging the HCMV-infected cells with γ-radiation led to bypassing the inhibitory effect of HCMV, resulting in remarkably higher apoptotic frequencies than were seen with either HCMV or radiation alone (1.8- *versus* 3.3-fold) among cases only, further suggesting potential dysregulation in apoptosis. HCMV has been reported to express cellular proteins (AKT and Bcl-2) that protect tumor cells from apoptosis as well as anti-apoptotic gene products from UL 36–38. The products encoded by these transcripts act as viral inhibitors [52]. In addition Skaletskaya *et al*. [53], reported the ability of HCMV to inhibit apoptosis by suppressing the activation of caspase-8. However, the capability of γ-radiation-induced DNA damage to consequently escape the inhibitory effects of HCMV on apoptosis has not yet been fully understood and will require further research. Gasper *et al*. [54], suggested that potential modulation of cell-signaling pathways might allow radiation to circumvent the inhibitory effect of HCMV on cell apoptosis. This study illustrates, most notably in cases, this potential and the need to further explore the effects that HCMV-radiated cells have on cell signaling modifications and changes. 

## 4. Conclusions

Overall, the data presented in the current study suggest that HCMV infection induces genomic instabilities comparable to ionizing radiation; a known risk factor for glioma development. Furthermore, our results indicate a significant increased sensitivity of PBLs to chromosomal aberrations in the presence of radiated HCMV cells. This study also demonstrates the comprehensive nature of the CBMN-Cyt assay and its ability to generate various measures of genomic instabilities via chromosomal breaks (MN) rearrangements (NPBs), as well as cell death (apoptosis). Its sensitivity and accuracy is invaluable in the design and execution of large scale epidemiological studies along a wide-spectrum of diseases. A limitation of our study is the lack of knowledge of the previous history of HCMV infection in the cases or controls, which may explain the baseline differences in genetic damage observed. Since HCMV infection is so prevalent in the population, future studies should adjust for genetic damage associated with previous infections. Future studies, aiming at exploring the role of the different DNA repair pathways in repair of HCMV-induced damage as compared to radiation damage, will further our understanding of the underlying mechanisms involved in any HCMV contributions to oncogenesis. 
